# Supplementary material for: Redox signaling-driven modulation of microbial biosynthesis and biocatalysis
Source: Nat Commun. 2023 Oct 26;14:6800. doi: 10.1038/s41467-023-42561-3 (PMC10603113; doi:10.1038/s41467-023-42561-3)
Supplement: Supplementary file 1 — Supplementary Information [file 41467_2023_42561_MOESM1_ESM.pdf]

Supporting Information

Redox signaling-driven modulation of microbial biosynthesis and biocatalysis

Na Chen[1,4], Na Du[1,4], Ruichen Shen[2], Tianpei He[1], Jing Xi[1], Jie Tan[2], Guangkai Bian[3], Yanbing Yang[1], Tiangang Liu[1], Weihong Tan[2], Lilei Yu[1*], Quan Yuan[1,2*]

[1]Renmin Hospital of Wuhan University, College of Chemistry and Molecular Sciences, Institute of Molecular Medicine, School of Microelectronics, School of Pharmaceutical Sciences, Wuhan University, Wuhan 430072, P. R. China.

[2]Molecular Science and Biomedicine Laboratory (MBL), State Key Laboratory of Chemo/Biosensing and Chemometrics College of Chemistry and Chemical Engineering, Hunan University, Changsha 410082, P. R. China.

[3]Center for Materials Synthetic Biology, Shenzhen Institute of Synthetic Biology, Chinese Academy of Sciences, Shenzhen 518055, P. R. China

[4]These authors contributed equally: Na Chen, Na Du.

*e-mail: yuanquan@whu.edu.cn; lileiyu@whu.edu.cn;

Contents

Supplementary Figures **4**
Supplementary Fig. 1| SEM images of *S. putrefaciens*. 4
Supplementary Fig. 2| Concentration of Fe^{2+} in the biological LAN with or without HA at different incubation time. Data presented as mean values ± SD, *n* = 3. 4
Supplementary Fig. 3| SEM images of *R. palustris*. 5
Supplementary Fig. 4| Schematic illustration of lycopene biosynthesis process with the MVA and MEP pathway[1,2]. 5
Supplementary Fig. 5| (a) Concentration of Fe^{2+} at different time in bare *S. putrefaciens* and microbial co-culture (*S. putrefaciens-R. palustris*) system. Data presented as mean values ± SD, *n* = 3. (b) OD_{600} of each culture over time. 6
Supplementary Fig. 6| Effects of inoculation ratios of the strains *R. palustris* and *S. putrefaciens* on lycopene biosynthesis efficiency (A represents *R. palustris* and B represents *S. putrefaciens*). Data presented as mean values ± SD, *n* = 3. 6
Supplementary Fig. 7| Relative concentration of NADPH in *R. palustris* isolated from the biological LAN with or without Fe redox communication. Data presented as mean values ± SD, *n* = 3. 7
Supplementary Fig. 8| (a) TEM image and (b) XRD pattern of ZGO:Mn nano verifier. 7
Supplementary Fig. 9| (a) Diagrammatic illustration of the energy level of ZGO:Mn nanorods[3] and Fe^{3+}/Fe^{2+}. (b) Persistent luminescence curves of ZGO:Mn nanorods in response to different substances. (c) Persistent luminescence recorded at different time. (d) Linear relationship between logarithm of persistent luminescence intensity and Fe^{2+}/Fe_{total} ratio. Data presented as mean values ± SD, *n* = 3. (e) Relative persistent luminescence intensity of ZGO:Mn nano verifier upon the addition of GSSG and GSH. Data presented as mean values ± SD, *n* = 3. (f) Relative persistent luminescence intensity of ZGO:Mn nano verifier in the supernatant of *S. putrefaciens-R. palustris* co-culture. Data presented as mean values ± SD, *n* = 3. 8
Supplementary Fig. 10| Relative CFU counts of (a) *S. putrefaciens* and (b) *R. palustris* incubated with the ZGO:Mn nano verifier. Data presented as mean values ± SD, *n* = 3. 8
Supplementary Fig. 11| Persistent luminescence intensity of ZGO:Mn nano verifier in bare *S. putrefaciens* and microbial co-culture system at different time. The initial concentrations of Fe^{3+} were both 2 mM in above two systems. Data presented as mean values ± SD, *n* = 3. 9
Supplementary Fig. 12| OD_{600} of the *S. putrefaciens-R. palustris* co-culture with or without Fe redox communication ("with Fe signal" represent the addition of Fe^{3+} with the initial concentration of 2 mM, "without Fe signal" represent no addition of Fe^{3+}). Data presented as mean values ± SD, *n* = 3. 9
Supplementary Fig. 13| OD_{600} of *S. putrefaciens* and *R. palustris* over incubation time in the biological LAN. Data presented as mean values ± SD, *n* = 3. 10
Supplementary Fig. 14| Calculated OD_{600} of the *S. putrefaciens* in *S. putrefaciens-R. palustris* co-culture across time. Data presented as mean values ± SD, *n* = 3. 10
Supplementary Fig. 15| Current densities of *S. putrefaciens-R. palustris* co-culture with and without redox communication (with or without Fe redox communication represented by "+"and "–" respectively). Data presented as mean values ± SD, *n* = 3. 11
Supplementary Fig. 16| Violin plots showing the significant differences in expression of (a) MVA & MEP related genes in *R. palustris* and (b) CO_2 fixation related genes. 11

Supplementary Fig. 17| A Volcano plot of differentially expressed genes in *S. putrefaciens*. The threshold of Log_2 FC is |0.58| (i.e., FC ≥ |1.5|), and that of *P* value is < 0.05. 12
Supplementary Fig. 18| Violin plots showing the significant differences in expression of (a) electron transfer related genes and (b) reducing equivalent related genes in *S. putrefaciens*. 12
Supplementary Fig. 19| Schematic illustration of iron oxidation pathway in *R. palustris*[4]. 13
Supplementary Fig. 20| Schematic illustration of how carbonyl cyanide m-chlorophenyl hydrazine (CCCP) block electron transport[5,6]. PMF represents the proton motive force formed by the electron carrier under the normal activity of the electron transport chain. The site of chemical inhibition is indicated by a red square on the electron path diagrams. 13
Supplementary Fig. 21| Schematic illustration of how rotenone block electron transport[5,6]. NADH-DH represents the NADH dehydrogenase. The site of chemical inhibition is indicated by a red halo on the electron path diagrams. 14
Supplementary Fig. 22| Lycopene yield of the biological LAN with Fe redox communication at different conditions. Data presented as mean values ± SD, *n* = 3. 14
Supplementary Fig. 23| (a) The Lycopene biosynthesis yield and OD_{600} across fermentation time in fed-batch fermentations (Inset: Photograph of the *S. putrefaciens*-*R. palustris* co-culture system with Fe redox communication for fed-batch fermentation). Data presented as mean values ± SD, *n* = 3. (b) Comparison of the lycopene biosynthesis yield between shake-flask fermentation and fed-batch fermentation after 96 h. Data presented as mean values ± SD, *n* = 3. (c) The lycopene biosynthesis yield of fed-batch fermentation in different batches. Data presented as mean values ± SD, *n* = 3. 15
Supplementary Fig. 24| Relative concentration of NADH in isolated *R. palustris* from *S. putrefaciens*-*R. palustris* co-culture. Data presented as mean values ± SD, *n* = 3. 15
Supplementary Fig. 26| Relative persistent luminescence intensity of ZGO:Mn nano verifier in the supernatant of *S. putrefaciens*-*R. palustris*-*G. Soli* microbial consortia. Data presented as mean values ± SD, *n* = 3. 16
Supplementary Tables 17
Supplementary Table 1| Primer used for *S. putrefaciens* mutants 17
Supplementary Table 2| Conditions used in model. 18
Supplementary Table 3| Constants used in model. 18
Supplementary References 19

Supplementary Figures

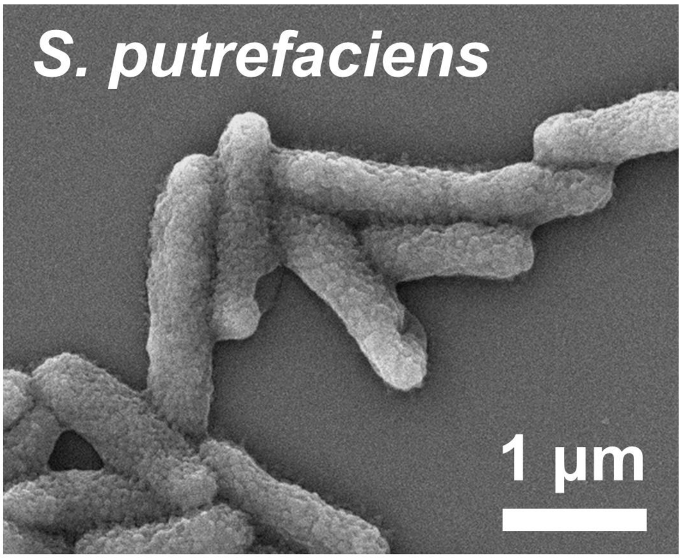

Supplementary Fig. 1| SEM images of *S. putrefaciens*.

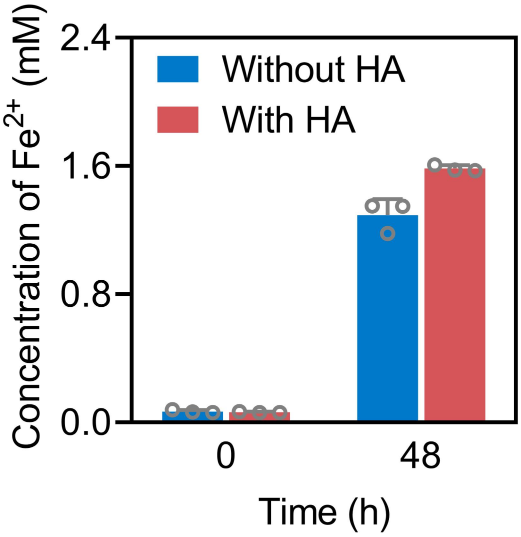

Supplementary Fig. 2| Concentration of Fe^{2+} in the biological LAN with or without HA at different incubation time. Data presented as mean values ± SD, $n = 3$.

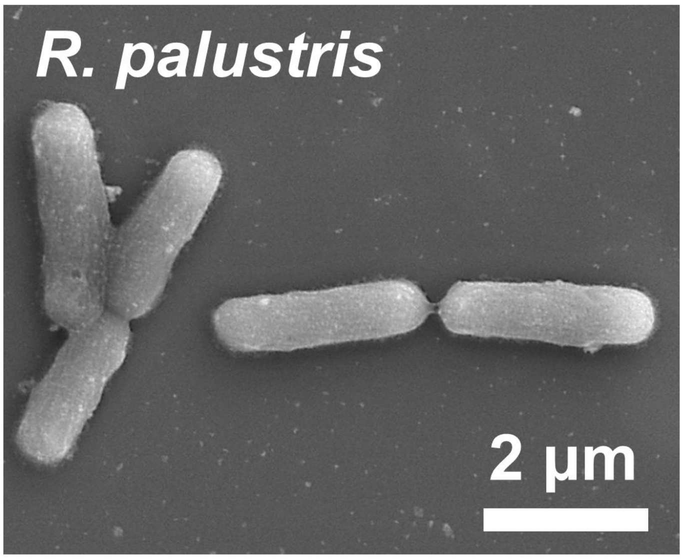

Supplementary Fig. 3| SEM images of *R. palustris*.

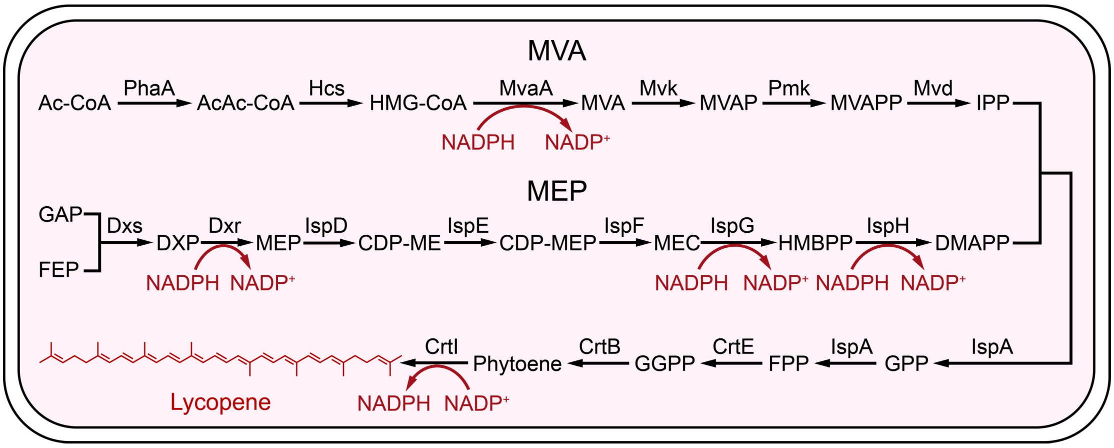

Supplementary Fig. 4| Schematic illustration of lycopene biosynthesis process with the MVA and MEP pathway[1,2].

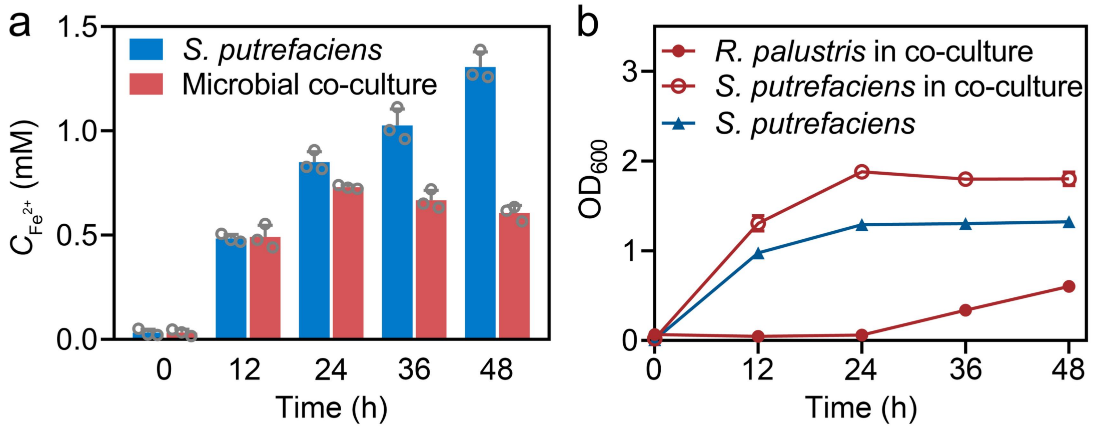

Supplementary Fig. 5| (a) Concentration of Fe^{2+} at different time in bare *S. putrefaciens* and microbial co-culture (*S. putrefaciens*-*R. palustris*) system. Data presented as mean values ± SD, *n* = 3. (b) OD_{600} of each culture over time.

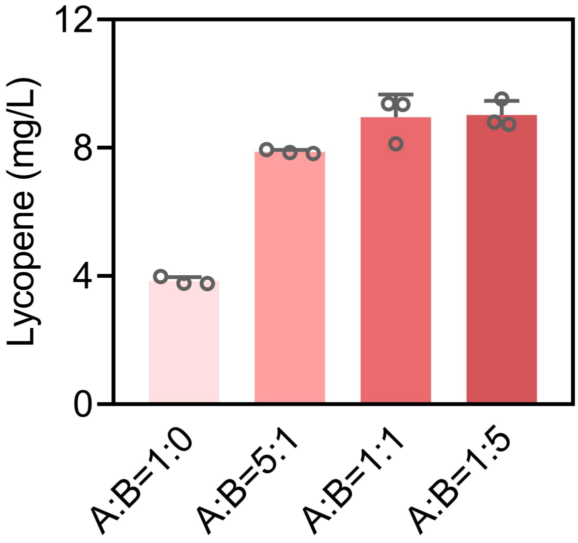

Supplementary Fig. 6| Effects of inoculation ratios of the strains *R. palustris* and *S. putrefaciens* on lycopene biosynthesis efficiency (A represents *R. palustris* and B represents *S. putrefaciens*). Data presented as mean values ± SD, *n* = 3.

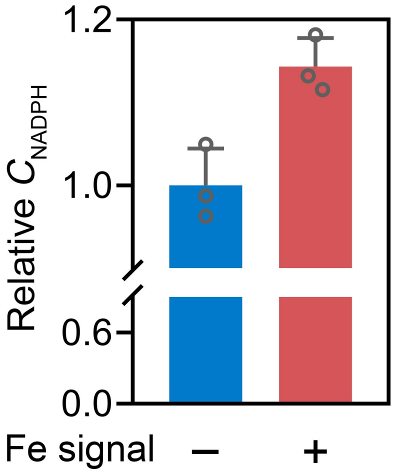

Supplementary Fig. 7| Relative concentration of NADPH in *R. palustris* isolated from the biological LAN with or without Fe redox communication. Data presented as mean values ± SD, $n = 3$.

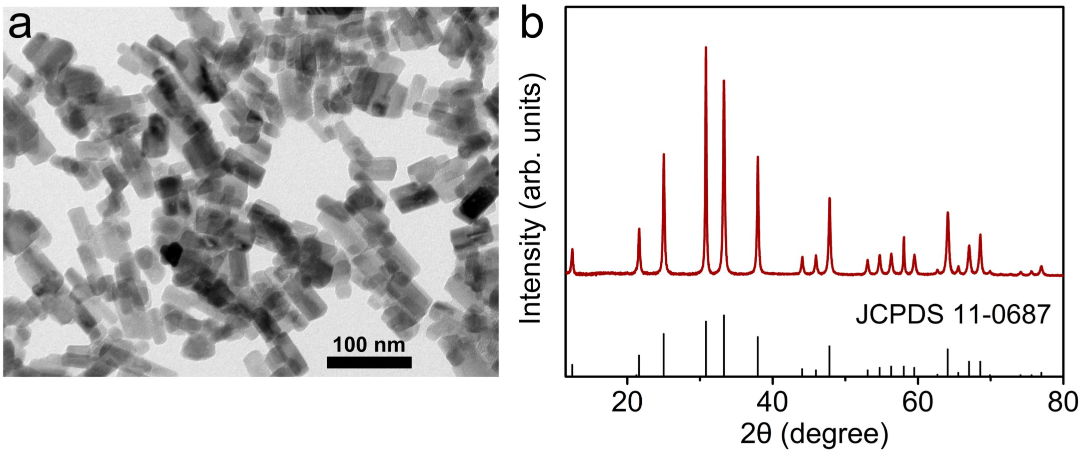

Supplementary Fig. 8| (a) TEM image and (b) XRD pattern of ZGO:Mn nano verifier.

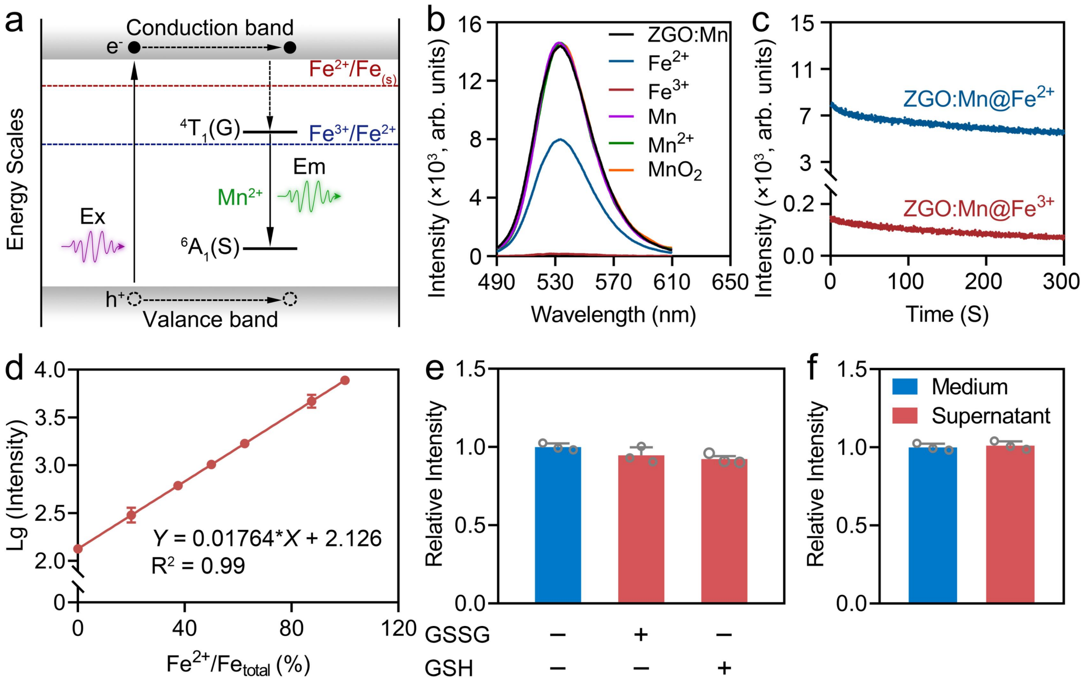

Supplementary Fig. 9| (a) Diagrammatic illustration of the energy level of ZGO:Mn nanorods[3] and Fe^{3+}/Fe^{2+}. (b) Persistent luminescence curves of ZGO:Mn nanorods in response to different substances. (c) Persistent luminescence recorded at different time. (d) Linear relationship between logarithm of persistent luminescence intensity and Fe^{2+}/Fe_{total} ratio. Data presented as mean values ± SD, n = 3. (e) Relative persistent luminescence intensity of ZGO:Mn nano verifier upon the addition of GSSG and GSH. Data presented as mean values ± SD, n = 3. (f) Relative persistent luminescence intensity of ZGO:Mn nano verifier in the supernatant of *S. putrefaciens*-*R. palustris* co-culture. Data presented as mean values ± SD, n = 3.

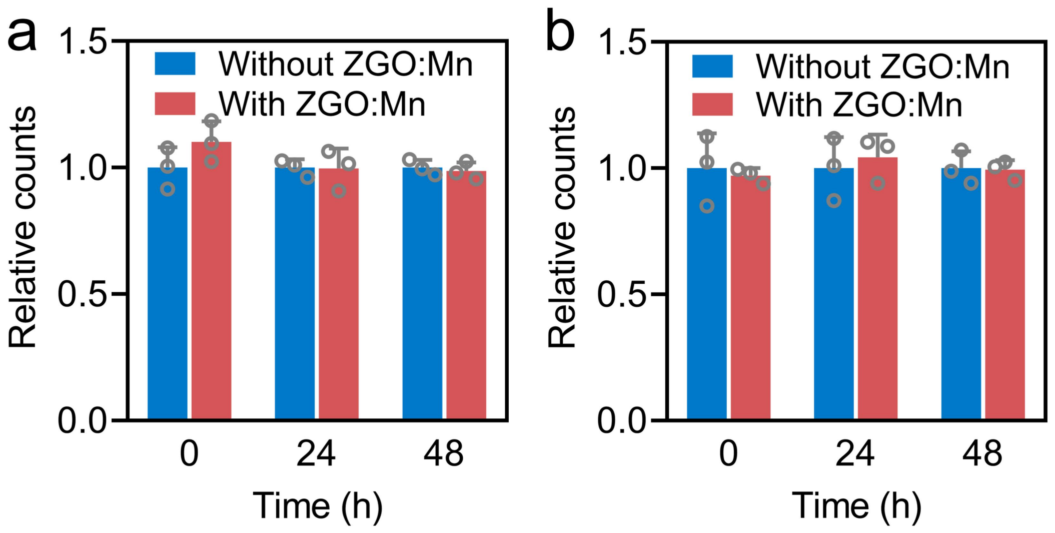

Supplementary Fig. 10| Relative CFU counts of (a) *S. putrefaciens* and (b) *R. palustris* incubated with the ZGO:Mn nano verifier. Data presented as mean values ± SD, n = 3.

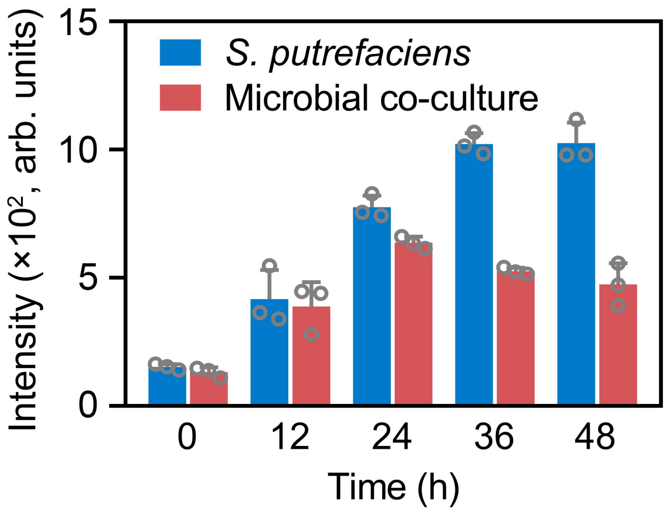

Supplementary Fig. 11| Persistent luminescence intensity of ZGO:Mn nano verifier in bare *S. putrefaciens* and microbial co-culture system at different time. The initial concentrations of Fe^{3+} were both 2 mM in above two systems. Data presented as mean values ± SD, *n* = 3.

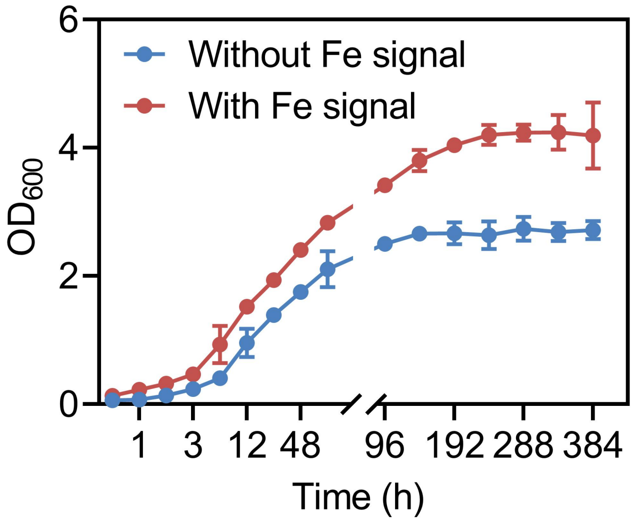

Supplementary Fig. 12| OD_{600} of the *S. putrefaciens-R. palustris* co-culture with or without Fe redox communication ("with Fe signal" represent the addition of Fe^{3+} with the initial concentration of 2 mM, "without Fe signal" represent no addition of Fe^{3+}). Data presented as mean values ± SD, *n* = 3.

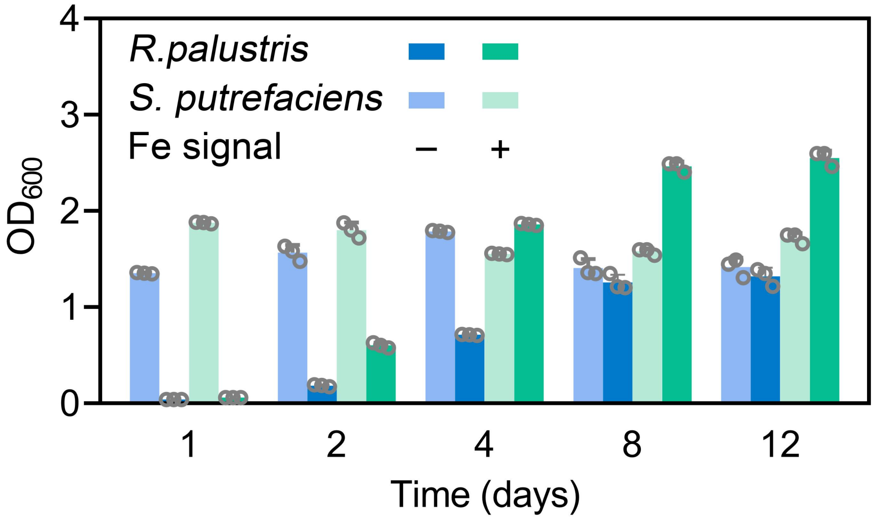

Supplementary Fig. 13| OD_{600} of *S. putrefaciens* and *R. palustris* over incubation time in the biological LAN. Data presented as mean values ± SD, *n* = 3.

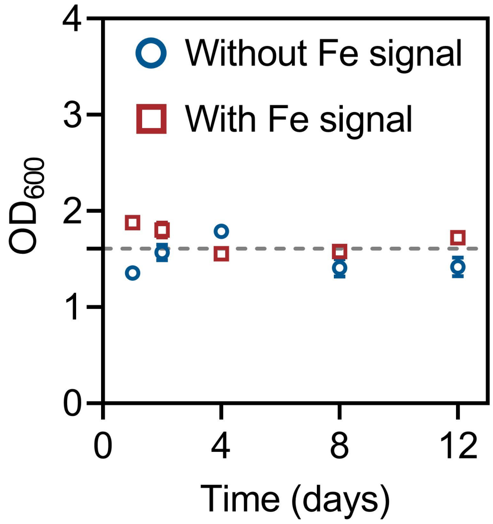

Supplementary Fig. 14| Calculated OD_{600} of the *S. putrefaciens* in *S. putrefaciens*-*R. palustris* co-culture across time. Data presented as mean values ± SD, *n* = 3.

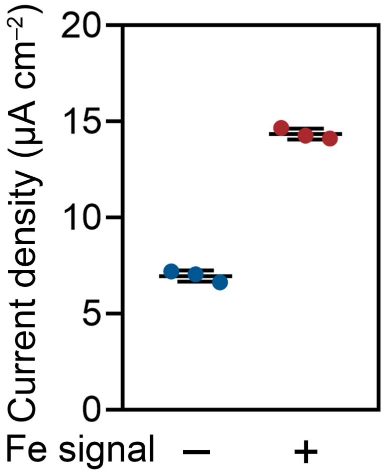

Supplementary Fig. 15| Current densities of *S. putrefaciens-R. palustris* co-culture with and without redox communication (with or without Fe redox communication represented by "+" and "–" respectively). Data presented as mean values ± SD, $n = 3$.

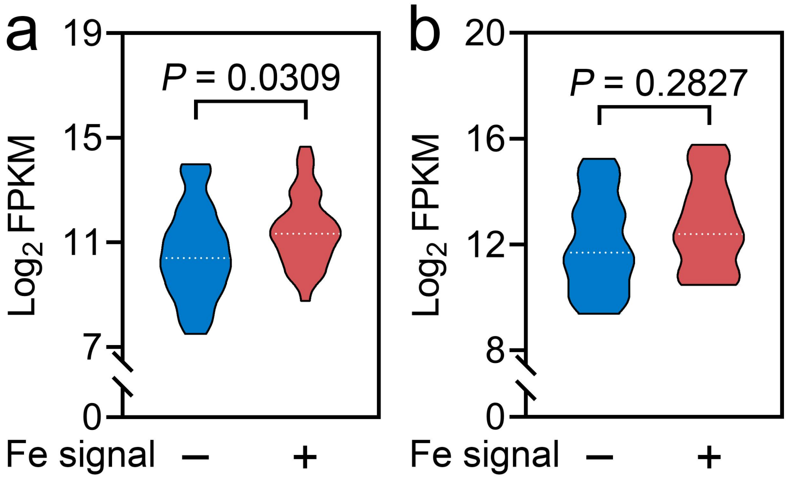

Supplementary Fig. 16| Violin plots showing the significant differences in expression of (a) MVA & MEP related genes in *R. palustris* and (b) CO_2 fixation related genes.

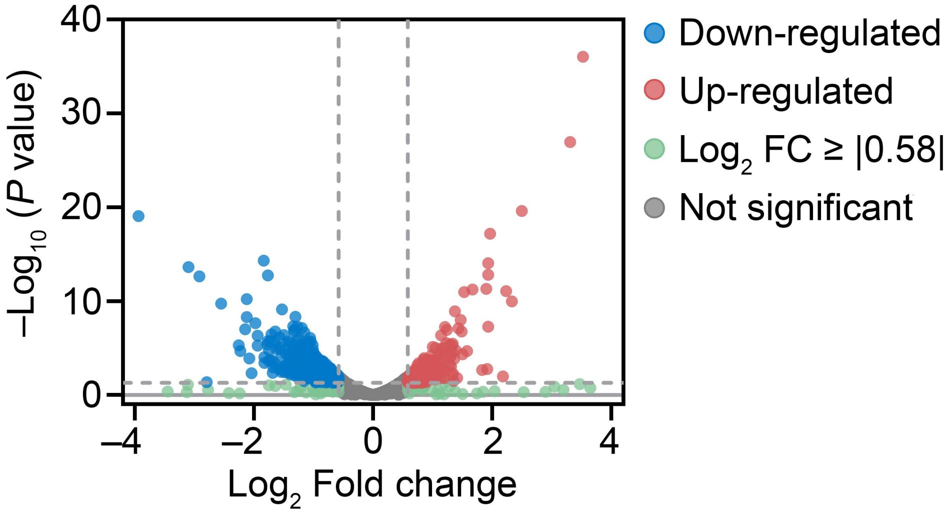

Supplementary Fig. 17| A Volcano plot of differentially expressed genes in *S. putrefaciens*. The threshold of Log_2 FC is |0.58| (i.e., FC ≥ |1.5|), and that of *P* value is < 0.05.

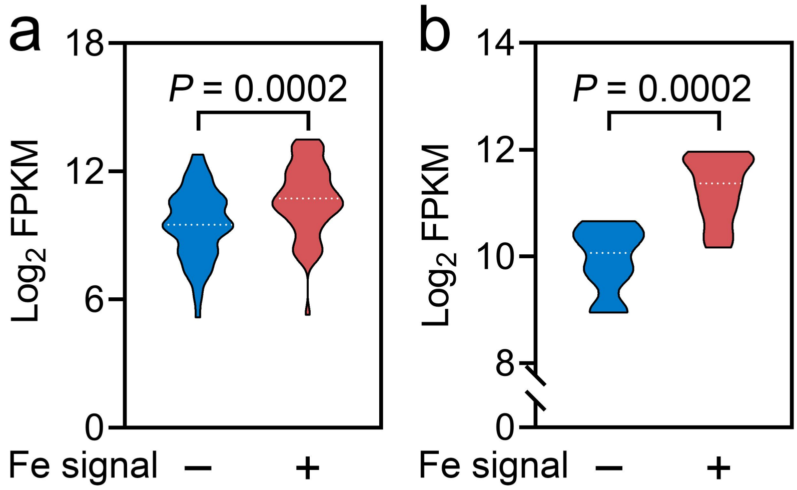

Supplementary Fig. 18| Violin plots showing the significant differences in expression of (a) electron transfer related genes and (b) reducing equivalent related genes in *S. putrefaciens*.

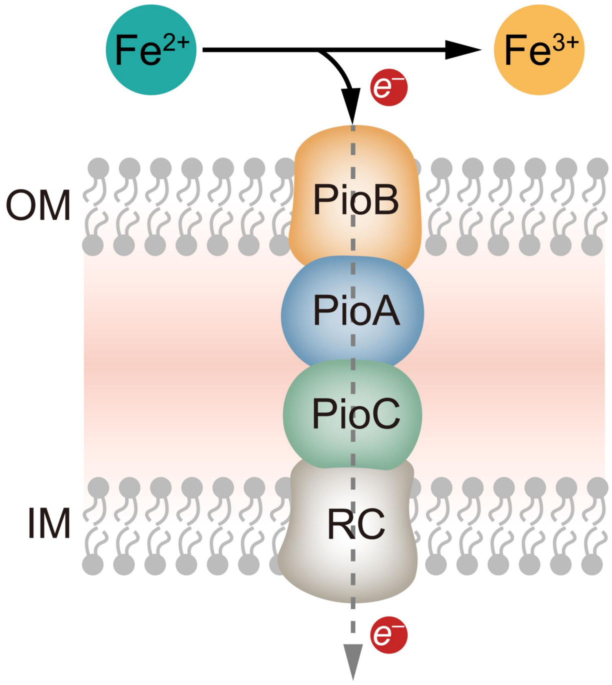

Supplementary Fig. 19| Schematic illustration of iron oxidation pathway in *R. palustris*[4].

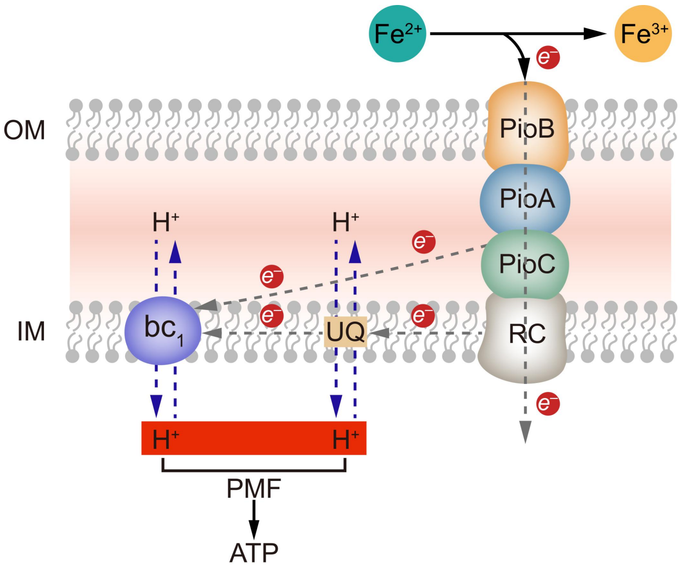

Supplementary Fig. 20| Schematic illustration of how carbonyl cyanide m-chlorophenyl hydrazine (CCCP) block electron transport[5,6]. PMF represents the proton motive force formed by the electron carrier under the normal activity of the electron transport chain. The site of chemical inhibition is indicated by a red square on the electron path diagrams.

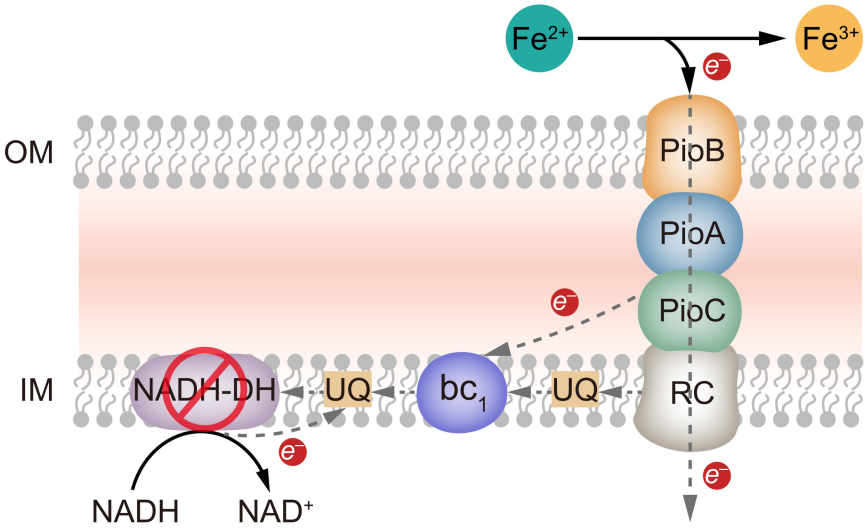

Supplementary Fig. 21| Schematic illustration of how rotenone block electron transport[5,6]. NADH-DH represents the NADH dehydrogenase. The site of chemical inhibition is indicated by a red halo on the electron path diagrams.

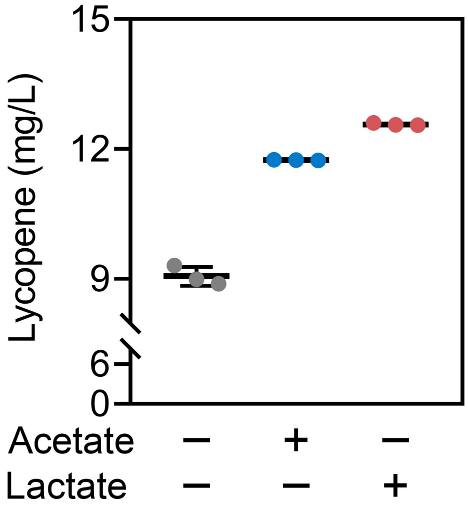

Supplementary Fig. 22| Lycopene yield of the biological LAN with Fe redox communication at different conditions. Data presented as mean values ± SD, $n = 3$.

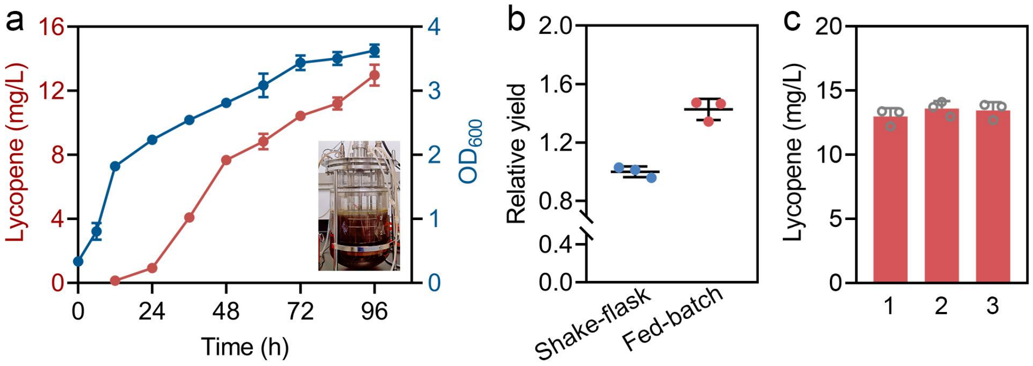

Supplementary Fig. 23| (a) The Lycopene biosynthesis yield and OD_{600} across fermentation time in fed-batch fermentations (Inset: Photograph of the *S. putrefaciens*-*R. palustris* co-culture system with Fe redox communication for fed-batch fermentation). Data presented as mean values ± SD, *n* = 3. (b) Comparison of the lycopene biosynthesis yield between shake-flask fermentation and fed-batch fermentation after 96 h. Data presented as mean values ± SD, *n* = 3. (c) The lycopene biosynthesis yield of fed-batch fermentation in different batches. Data presented as mean values ± SD, *n* = 3.

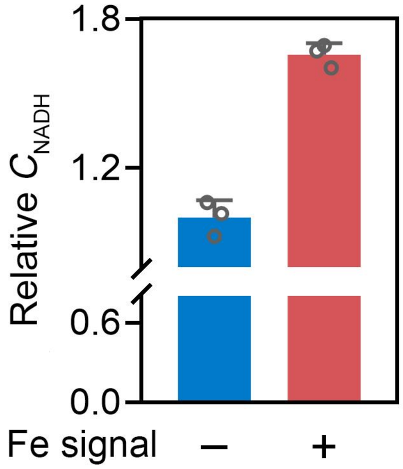

Supplementary Fig. 24| Relative concentration of NADH in isolated *R. palustris* from *S. putrefaciens*-*R. palustris* co-culture. Data presented as mean values ± SD, *n* = 3.

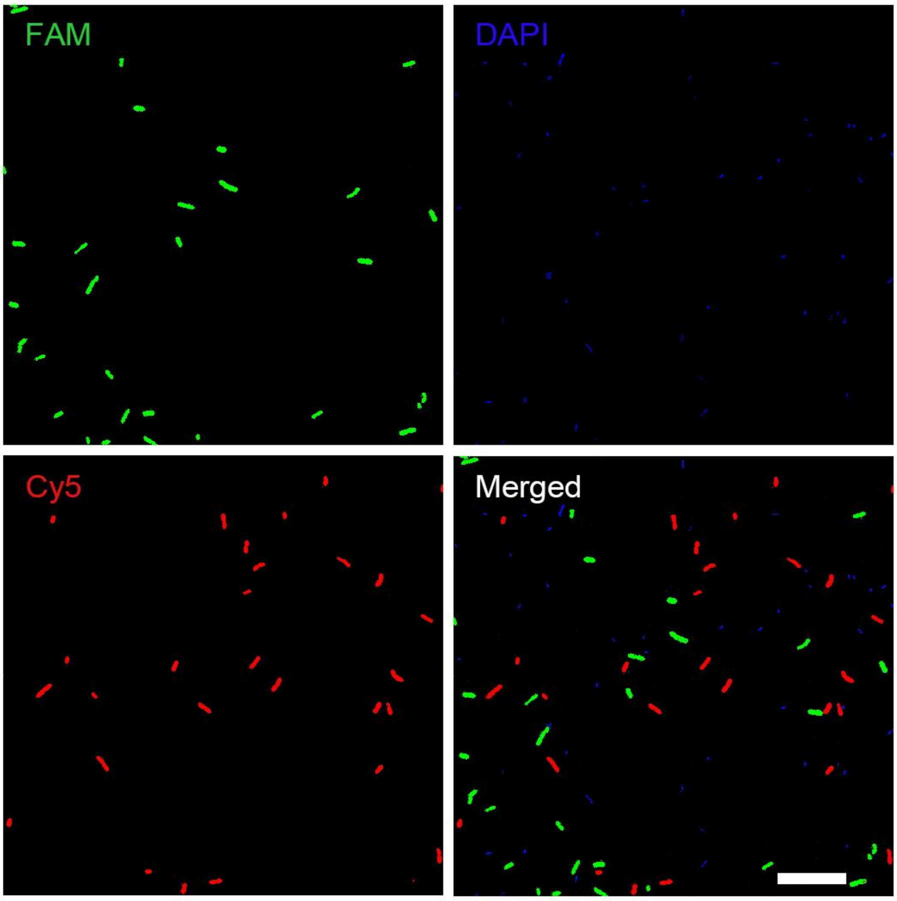

Supplementary Fig. 25| Confocal fluorescence imaging of *S. putrefaciens* (FAM, green), *G. Soli* (DAPI, blue) and *R. palustris* (Cy5, red) in microbial consortia. Scale bar is 5 μm. At least three independent experiments were carried out with similar results.

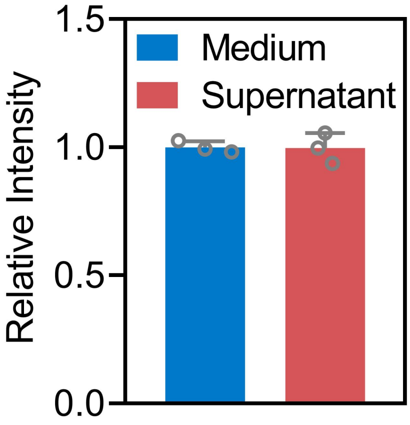

Supplementary Fig. 26| Relative persistent luminescence intensity of ZGO:Mn nano verifier in the supernatant of *S. putrefaciens*-*R. palustris*-*G. Soli* microbial consortia. Data presented as mean values ± SD, $n = 3$.

Supplementary Tables

Supplementary Table 1| Primer used for *S. putrefaciens* mutants

Primer	Sequence	Function
CymA-UP-samI-F	TCCCCCGGGTTGAGCATGATGTGGTGCCATC	*CymA* deletion
CymA-down-kpnI-R	GGGGTACCACGCTGACACTCTAGTGGTGTT	*CymA* deletion
CymA-UP-R	GAATCGCTAAAACCTATTACTCTATCTCCAAAATTGCC	*CymA* deletion
CymA-down-F	AATTTTGGAGATAGAGTAATAGGTTTTAGCGATTCATTC	*CymA* deletion
CymA-JD-F	TCGCACTCATATCCATAGTGT	△*CymA* verification
CymA-JD-R	GCTGTTTAAGAAATTACTGCC	△*CymA* verification
Mtrc-UP-smaI-F	TCCCCCGGGGACGCAATTTTCAGTAACGG	*Mtrc* deletion
Mtrc-down-kpnI-R	GGGGTACCTAGCAGTTGTCATACATCACAA	*Mtrc* deletion
Mtrc-UP-F	ACCTATGCAGGGAAAAATTTGCCCAAGCGGGGGGAGTCTA	*Mtrc* deletion
Mtrc-UP-R	TCCCCCCGCTTGGGCAAATTTTTCCCTGCATAGGTTTGGCA	*Mtrc* deletion
Mtrc-JD-F	CACCCTTATTGTGATTCCCC	△*Mtrc* verification
Mtrc-JD-R	GCCCTTATGTTTAACTACCTC	△*Mtrc* verification

Supplementary Table 2| Conditions used in model.

Species	Description
$[Fe^{2+}]_{S'}$	Fe^{2+} concentration in bare *S. putrefaciens* system (mM)
$[Fe^{2+}]_{R'}$	Fe^{2+} concentration in bare *R. palustris* system (mM)
$[Fe^{2+}]_{S}$	Reduced Fe^{2+} concentration by *S. putrefaciens* in the biological LAN (mM)
$[Fe^{2+}]_{R}$	Oxidized Fe^{2+} concentration by *R. palustris* in the biological LAN (mM)
$[Cells]_S$	OD_{600} value of *S. putrefaciens* in the biological LAN (OD_{600})
$[Cells]_R$	OD_{600} value of *R. palustris* in the biological LAN (OD_{600})
t	Time (hour)

Supplementary Table 3| Constants used in model.

Parameter	Value	Parameter	Value
A_S	0.0175	A_R	-0.00504
B_S	1.04587	B_R	0.57264
C_S	10.9096	C_R	12.64864
D_S	1.90874	D_R	1.42455
E_S	0.735	E_R	0.132
F_S	326.59067	F_R	102.38697
G_S	-0.99948	G_R	0.06048
H_S	66.79425	H_R	67.0344
I_S	3..21653	I_R	3.01761
J_S	0.259	J_R	1.18
K_S	16921.27315	K_R	16734.13732
L_S	3.81207	L_R	90.70119
M_S	128.01808	M_R	125.55255
N_S	5.11874	I_R	5.6497

Supplementary References

1. Li, M. et al. Enhanced CO_2 capture for photosynthetic lycopene production in engineered *Rhodopseudomonas Palustris*, a purple nonsulfur bacterium. *Green Chem.* **24**, 7500–7518 (2022).
2. Li, M., Xia, Q., Zhang, H., Zhang, R. & Yang, J. Metabolic engineering of different microbial hosts for lycopene production. *J. Agric. Food. Chem.* **68**, 14104–14122. (2020).
3. Chen, N. et al. Real-time monitoring of dynamic microbial Fe(III) respiration metabolism with a living cell-compatible electron-sensing probe. *Angew. Chem. Int. Ed.* **61**, e202115572 (2022).
4. Shi, L. et al. Extracellular electron transfer mechanisms between microorganisms and minerals. *Nat. Rev. Microbiol.* **14**, 651–662 (2016).
5. Guzman, M. S. et al. Phototrophic extracellular electron uptake is linked to carbon dioxide fixation in the bacterium *Rhodopseudomonas Palustris*. *Nat. Commun.* **10**, 1355 (2019).
6. Liu, X. et al. Syntrophic interspecies electron transfer drives carbon fixation and growth by *Rhodopseudomonas Palustris* under dark, anoxic conditions. *Sci. Adv.* **7**, eabh1852 (2021).
